# Impact of PCI strategies on outcomes of patients undergoing Transcatheter Aortic Valve Implantation with concomitant coronary artery disease: A systematic review and meta-analysis

**DOI:** 10.1371/journal.pone.0321395

**Published:** 2025-04-30

**Authors:** Dayang Wang, Sijia Lai, Zichen Wang, Changbo Xuan, Xiaoxia Ren, Wenhua Peng, Guozhong Pan

**Affiliations:** 1 Institute of Cardiovascular Diseases, Dongzhimen Hospital, Beijing University of Chinese Medicine, Beijing, China; 2 Second Department of Cardiology, Dongzhimen Hospital, Beijing University of Chinese Medicine, Beijing, China.; 3 Beijing Hospital of Traditional Chinese Medicine, Beijing, China; Saud Al-Babtain Cardiac Centre, SAUDI ARABIA

## Abstract

The aim of this study is to compare the clinical benefits associated with different percutaneous coronary intervention (PCI) timing strategies in patients undergoing transcatheter aortic valve implantation (TAVI) who have coexisting coronary artery disease (CAD). A systematic review and meta-analysis were conducted. PubMed, EMBASE, Cochrane Library and Web of Science databases were searched for relevant articles up to April 10th, 2024. Studies that reported comparisons of clinical outcomes between PCI before/concomitant with TAVI (PCI-TAVI) vs. TAVI alone, or comparisons between PCI before/concomitant with TAVI vs. PCI after TAVI (TAVI-PCI) were selected. Primary outcomes were all-cause mortality in the short-term, mid-term and long-term follow-up. A total of 23 studies pooling 15812 patients were included. Compared to TAVI alone, PCI-TAVI showed no significant difference in all-cause mortality at short- and mid-term (RR_short-term_ = 1.10 95%CI 0.88-1.38; RR_mid-term_ = 1.12 95%CI 0.97-1.30), but an increase during long-term follow-up (RR_long-term_ = 1.20 95%CI 1.06-1.36). Compared with PCI-TAVI, TAVI-PCI is associated with lower rate of all-cause mortality at both short- and long-term follow-ups. PCI before or concomitant with TAVI may not offer clinical benefits and could potentially lead to worse outcomes in the long term. Conversely, PCI after TAVI is associated with improved clinical outcomes in both the short and long term.

## Introduction

In patients scheduled to undergo Transcatheter Aortic Valve Implantation (TAVI), the prevalence of concomitant coronary artery disease (CAD) exceeds 65% [1–3]. Current guidelines recommend percutaneous coronary intervention (PCI) of coronary artery lesions with > 70% stenosis in proximal segments or > 50% in left main arteries in patients scheduled for TAVI [4]. Considerations underpinning guideline recommendation encompass: the deployment of the transcatheter heart valve (THV) platform may potentially exacerbate of the degree of stenosis in pre-existing proximal coronary lesions; THV platform may escalate the complexity of unscheduled coronary interventions (in patients experiencing concurrent myocardial infarction, obstruction of the coronary access can be fatal); severe coronary stenosis may increase the risk of serious complications during rapid ventricular pacing. Consequently, for safety reasons, administering PCI prior to TAVI constitutes standard clinical practice [5].

Nonetheless, prevailing evidence indicates that PCI lacks prognostic benefits in patients with stable CAD [6]. Currently, there is a paucity of clinical evidence supporting the improvement of prognosis through “TAVI-driven” PCI. Compared to TAVI, PCI requires longer anti-thrombotic therapy duration afterwards, elevating bleeding risk in TAVI patients; undertaking PCI before or concomitant with TAVI entails heightened contrast exposure, with an associated risk of potential acute kidney injury. Therefore, there is an ongoing debate regarding the potential benefits of performing PCI before or concomitantly with TAVI.

In recent years, there has been a gradual accumulation of clinical evidence addressing PCI strategies choice in this population. Findings from the SURTAVI study [7] revealed no substantial difference in long-term mortality between PCI concomitant with TAVI and TAVI alone. Similarly, the ACTIVITION trial [8] produced analogous outcomes while highlighting an elevated risk of bleeding associated with TAVI combined with PCI. The REVASC-TAVI study [5] showed that performing PCI after TAVI could potentially reduce both short and long-term mortality compared to PCI pre/ concomitant with TAVI, focusing on following established indication of PCI.

In light of the aforementioned controversies, we conducted a systematic review and meta-analysis aiming at evaluating the relationship between PCI timing strategies and clinical outcomes in patients undergoing TAVI with coexisting CAD.

## Methods

The protocol of this studies was registered with PROSPERO (NO. CRD42023459377). The research reported in this paper adhered to the Preferred Reporting Items for a Systematic Review and Meta-analysis (PRISMA) 2020 update [9].

### Data sources and searches

The literature searches were conducted in the following four databases: PubMed, EMBASE, Cochrane Library and Web of Science. The publication time was set from the inception to April 10th, 2024. We used the following subject terms and free words to perform search: “transcatheter aortic valve implantation”, “percutaneous coronary intervention”, “death”, “cardiovascular events”, “coronary artery disease” etc. S1 Table in the supplementary material showed search strategies in detail.

### Eligibility criteria, study selection and data extraction

Studies were considered eligible for inclusion if they met following criterion: 1.were published as a full-length article; 2.were written in English; 3.were randomized controlled trial(RCT) or cohort study (including prospective and retrospective cohorts); 4. not involving patients without CAD; 5.included comparisons between PCI before/concomitant with TAVI(PCI-TAVI) vs. TAVI alone, or comparisons between PCI before/concomitant with TAVI(PCI-TAVI) vs. PCI after TAVI(TAVI-PCI); 6.reported risk ratio (RR), adjusted hazard ratio (HR), or raw data of case number of all-cause mortality. Studies were eligible regardless of the type of THV used for TAVI.

Two independent reviewers (DW and SL) scanned titles and abstracts according to the inclusion criteria, reviewed full-text articles, and determined their eligibility. Missing data was requested from the corresponding author of included articles. Any discrepancy regarding searches and selection was discussed in consultation with and resolved by a third reviewer (GP). The full text was retrieved for further inspection if a study potentially met the inclusion criteria.

Two reviewers independently conducted data extraction. The data included: 1. study-level general information; 2. baseline characteristic of population; 3. outcomes from original eligible sources, including all-cause mortality, major adverse cardiovascular events (MACE), cardiovascular death, non-fatal myocardial infarction, stroke, or safety events (major bleeding, major access related complications or acute kidney injury). The ascertainment of clinical events was accepted as reported. According to the time of follow-up, we categorize short-term as 30 days, mid-term as 6 months to 1 year, and long-term as 2 years or longer. Discrepancy, if any, was verified and resolved by a third reviewer (GP). Records of studies were managed with the Endnote X9 software.

### Quality assessment

The risk of bias in cohort studies was assessed using the Newcastle-Ottawa Scale (NOS) [10]. Specifically, we stipulated that a follow-up duration of at least 1 year would be scored to account for the occurrence of death or other events. The methodological Quality of RCTs was assessed using the “Revised Cochrane Risk of Bias Tool for Randomized Trials” (RoB-2) [11].

### Statistical analysis

Meta-analyses were conducted for comparable studies using RevMan 5.4.1 software [12]. Summary effect size for all independent events were calculated using pooled RR. Data presented in form of the number of cases or hazard ratio, were converted to RR and 95% confidence interval (95%CI) utilizing the embedded calculation program within software. A random-effects model was used in all the outcomes. We did not conduct pooled analyses for outcomes reported in fewer than three studies. Inter-study heterogeneity was assessed using the I^2^ statistic, which was defined as I^2^ values of 50% or greater. The z statistic was computed for each outcome of interest, and the results were considered statistically significant at 1-sided *p* < 0.05. Meta-analysis results were presented using forest plots.

Subgroup analyses were performed to investigate possible sources of heterogeneity and to assess the effect of specific variables on results, including THV type (whether the proportion using SEV exceeded 20% among all patients who underwent TAVI included in study). To evaluate the robustness, we performed a sensitivity analysis for the outcome of all-cause mortality. This involved sequentially excluding individual studies to ascertain their impact on the total results.

The publication bias was assessed using funnel plots by displaying individual study effect for the outcomes of interest. Funnel plot asymmetry was also evaluated using Egger’s test (with 1-sided *p* <  0.1 indicating significant publication bias). The publication bias assessments were conducted using STATA 14 [13] software.

## Results

### Study selection and characteristics

**Fig 1:**
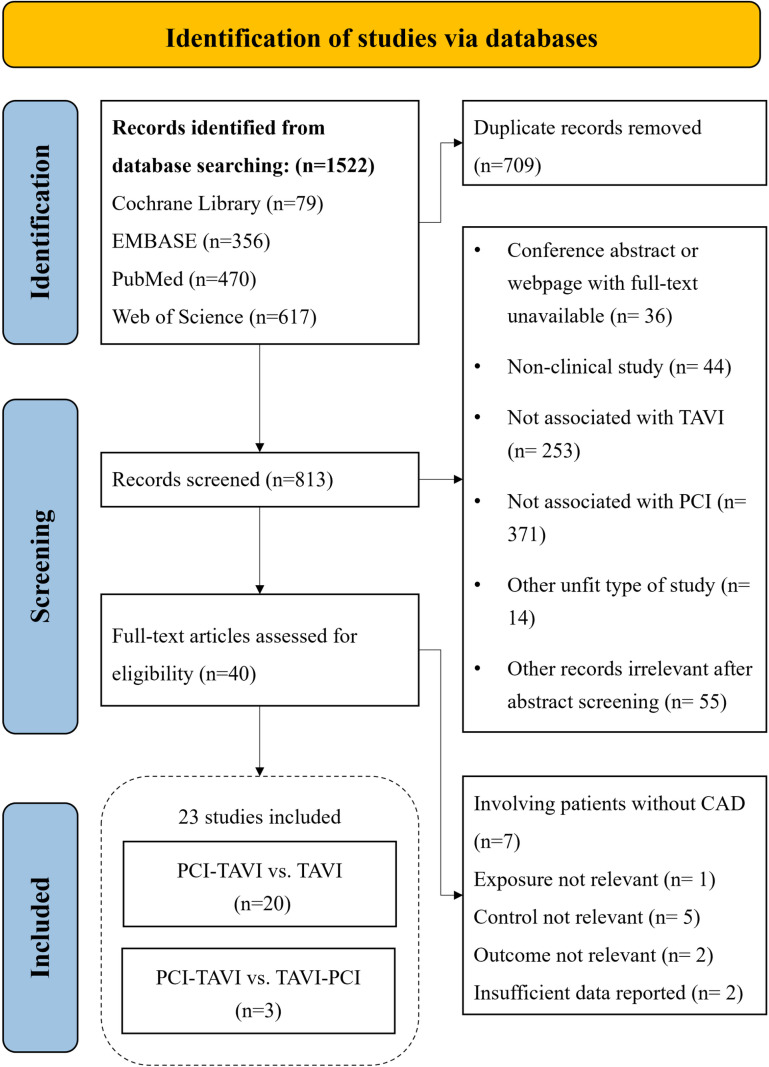
Flow chart of study selection process.

From the initial search, we identified 1522 studies, of which 709 duplicates were removed. Of the 813 remaining records, 773 were excluded after screening titles and abstracts due to irrelevance with TAVI or PCI, or inappropriate article types. After assessing the full text of the remaining 40 articles, we excluded 7 for involving patients without CAD, 5 for irrelevant control, 1 for irrelevant exposure, 2 for irrelevant outcomes, and 2 for insufficient data reported. Ultimately, thirty-three studies met the inclusion criteria and were included in the systematic review. Among them, twenty articles compared the clinical outcomes of PCI-TAVI and TAVI alone [8, 14–32]; three articles compared the clinical outcomes of PCI-TAVI and TAVI-PCI [33–35]. Flow chart of the study selection process is shown in Fig 1. Detailed reasons for exclusion are shown in S2 File.

In the comparison of PCI-TAVI vs. TAVI alone, a total of 13010 patients were included. One study was RCT, twenty-two were cohort studies. Ten types of THV were encompassed, including balloon-expandable valve (BEV) represented by Edward SAPIEN series; and self-expanding valve (SEV) represented by CoreValve series, Accurate etc. In the comparison of PCI-TAVI and TAVI-PCI, three studies involving 2802 patients were included. All these studies were cohort studies with 2 retrospective and 1 prospective. All three studies reported outcome of all-cause mortality at 30-days and 2-years follow-up. The general information of all included studies was displayed in Table 1. The average age of the included studies was 79.7 years, with women accounting for 46.9%. Detailed baseline characteristics are shown in Table 2.

**Table 1:** General characteristic of included studies.

Study ID	Country	Number of Patients	THV Type (self-expanding%)	Follow-up Period (year)	Reported Outcomes	Study Design
**Studies comparing PCI-TAVI vs. TAVI alone**						
Abramowitz 2014	Israel	144	NR	3	①②③④⑦⑧⑨	Prospective
Griese 2014	Germany	411	8.52%	2	①②③④⑥⑦⑧⑨	Prospective
Khawaja 2015	UK	93	0%	1	①②	Retrospective
Mancio 2015	Portugal	46	82.61%	2	①②	Prospective
Penkalla 2015	Germany	308	0%	5	①②	Retrospective
Huczek 2018	Poland	462	64.00%	0.1	①②	Retrospective
Millan-Iturbe 2018	Denmark	224	84.70%	9	①②⑤	Retrospective
Guedeney 2019	UK, France, Germany, Italy, Spain, Netherland	328	46.95%	1	①②③④⑥⑦⑧⑨	Prospective
Landt 2019	Germany	873	34.30%	1	②③④⑦⑥⑧⑨	Retrospective
Elbaz 2020	Canada	888	31.60%	1	①②	Retrospective
Karaduman 2021	Turkey	127	0%	1	①②⑥⑦⑨	Retrospective
Boogert 2021	Netherlands	577	18.20%	3	①②③④⑦	Retrospective
Matta 2021	France	372	41.70%	0.1	①②⑥⑦⑧⑨	Retrospective
Stephan 2021	Germany	333	NR	1	①②④⑤⑥	Retrospective
Minten 2022	Belgium	346	NR	5	②③⑥	Prospective
Patterson 2022	UK	235	<7.87%	1	①②③④⑥⑦⑧	RCT
Valvo 2023	Italy	252	NR	3	①②④⑥⑦⑧	Retrospective
Aurigemma 2023	Italy	1130	NR	1	①②④⑤⑥⑦⑨	Prospective
Mosleh 2023	USA	1809	26.30%	1	①②④⑤⑥⑦⑨	Retrospective
Khan 2024	USA	4052	NR	1	②④⑤⑦	Retrospective
**Studies comparing PCI before or concomitant with TAVI vs. PCI after TAVI.**						
Ochiai 2020	USA	258	12.66%	2	①②④⑥⑦⑧	Retrospective
Lunardi 2022	Italy	142	22.90%	2	①②③④⑥⑦⑧⑨	Retrospective
Rheude 2023	30 centers in Europe, North and South America and Japan	2402	56.43%	2	①②④⑥⑦⑧	Prospective

Outcomes Reported: ①Major adverse cardiovascular events. ②all-cause mortality. ③Cardiovascular Death. ④acute myocardial infarction. ⑤revascularization. ⑥Stroke. ⑦Major Bleeding. ⑧Acute kidney injury. ⑨Major Access-related complications. Abbreviations: THV=transcatheter heart valve. RCT=randomized controlled trial. NR=not reported. PCI=percutaneous coronary intervention. TAVI=transcatheter aortic valve implantation.

**Table 2.** Baseline characteristics of included studies.

Study ID	Age, year	Sex, female%	HT%	DM%		Previous Stroke%		Smoking%	LVEF%
**Studies comparing PCI-TAVI vs. TAVI alone**									
Abramowitz 2014	83.31	50.70	84.72	30.54		8.32		NR	54.95
Griese 2014	82.00	62.76	NR	35.26		10.45		NR	53.68
Khawaja 2015	83.80	52.00	58.00	19.00		13.00		NR	49.30
Mancio 2015	79.00	30.40	89.10	41.30		28.30		NR	47.00
Penkalla 2015	81.49	64.64	NR	32.17		23.99		NR	51.23
Huczek 2018	79.72	47.83	71.45	37.21		12.56		NR	52.71
Millan-Iturbe 2018	79.79	37.49	65.65	24.57		14.74		NR	NR
Guedeney 2019	82.62	100.00	81.74	31.10		7.63		3.38	55.00
Landt 2019	80.60	54.10	87.17	27.53		12.29		NR	53.40
Elbaz 2020	83.80	42.35	94.80	44.25		3.15		NR	NR
Boogert 2021	82.63	49.75	79.74	31.41		10.89		NR	NR
Karaduman 2021	78.94	55.89	89.74	33.85		8.66		NR	55.46
Matta 2021	84.00	47.29	68.03	29.04		11.05		1.60	56.89
Stephan 2021	81.40	52.00	NR	29.40		12.60		NR	61.70
Minten 2022	81.80	40.50	85.80	28.90		12.40		NR	49.90
Patterson 2022	83.95	38.69	78.31	26.39		3.81		3.81	NR
Valvo 2023	80.68	53.30	89.10	53.00		8.90		NR	52.94
Aurigemma 2023	82.10	49.00	NR	28.80		NR		NR	53.9
Mosleh 2023	81.87	36.5	90.75	37.73		12.00		20.03	55.52
Khan 2024	79.4	38.2	82.70	39.50		NR		NR	54.35
**Studies comparing PCI before or concomitant with TAVI vs. PCI after TAVI.**									
Ochiai 2020	81.29	29.62	100.00	89.84	31.66		14.58	NR	53.52
Lunardi 2022	81.43	53.00	100.00	70.31	26.21		6.04	NR	52.88
Rheude 2023	82.00	41.10	100.00	85.60	32.20		8.50	NR	58.00

Abbreviations: NR=not reported. PCI=percutaneous coronary intervention. TAVI=transcatheter aortic valve implantation.

### Quality assessment

**Fig 2:**
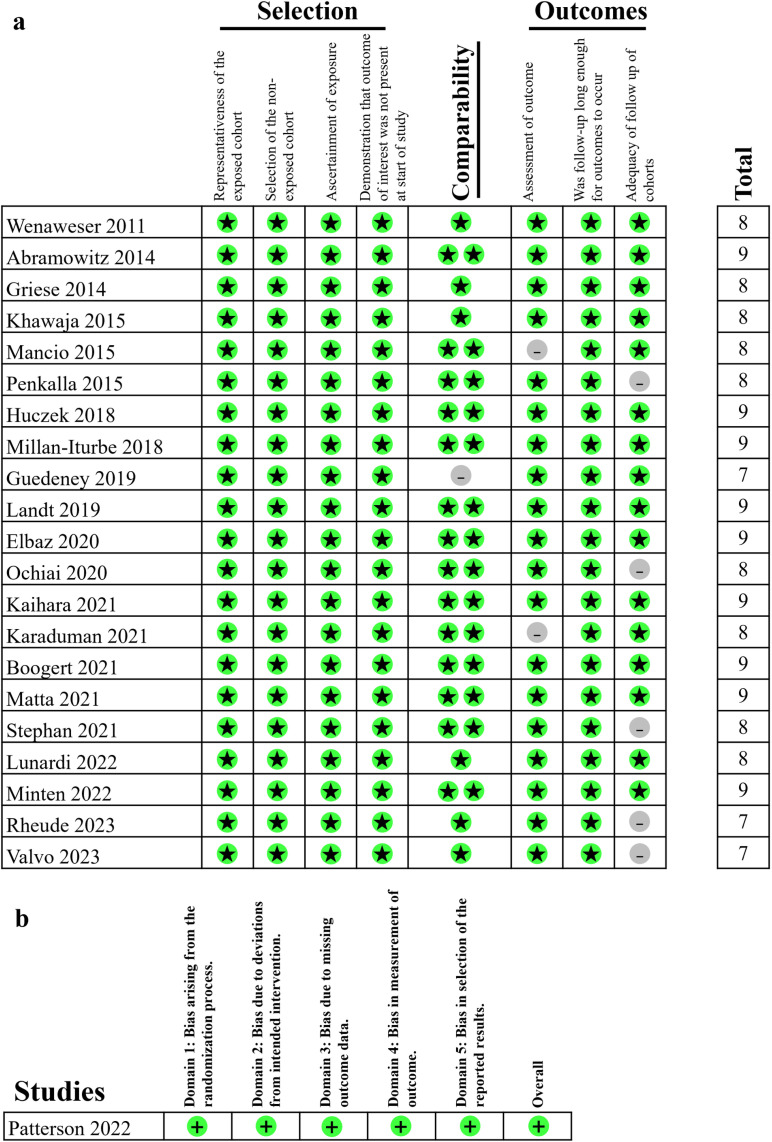
Risk of Bias Assessment of all included studies. a. The risk of bias for cohort studies assessed by NOS. b. The risk of bias for RCTs assessed by RoB-2 tool.

For observational studies, NOS tool was used to evaluate the methodologic quality. All twenty-two cohort studies were regarded as “good quality”. For randomized clinical trials, RoB-2 tool was used to evaluate the methodologic quality. The one RCT included in our meta-analysis were regarded to have “low risk of bias”. Details of quality assessment for individual studies using NOS and RoB-2 tools are shown in Fig 2.

### Outcomes

#### All-cause mortality.

**Fig 3:**
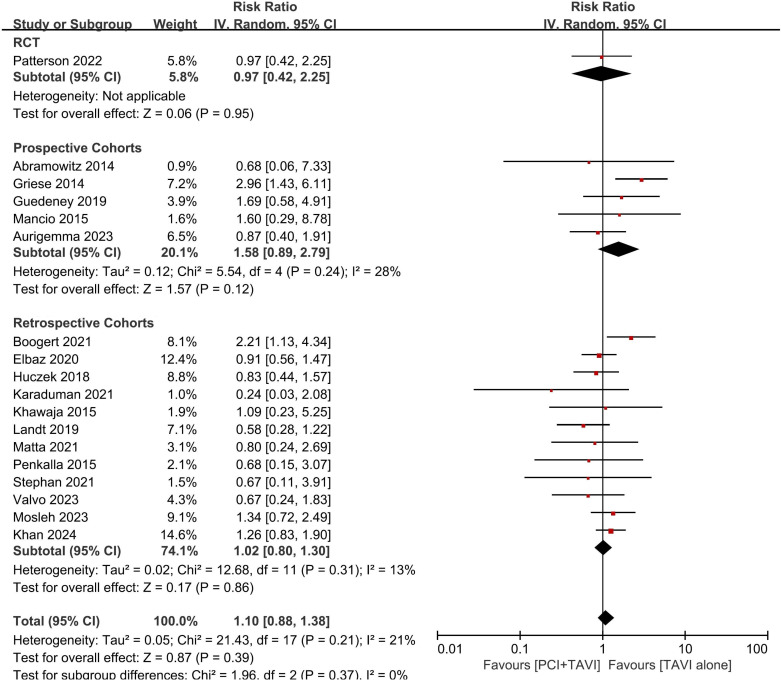
Forest plot of all-cause mortality at 30 days of patients undergoing TAVI plus PCI versus TAVI alone. Studies were stratified by study type. Abbreviations: CI=confidence interval. RCT=randomized controlled trial. TAVI=transcatheter aortic valve implantation. PCI=percutaneous coronary intervention.

**Fig 4:**
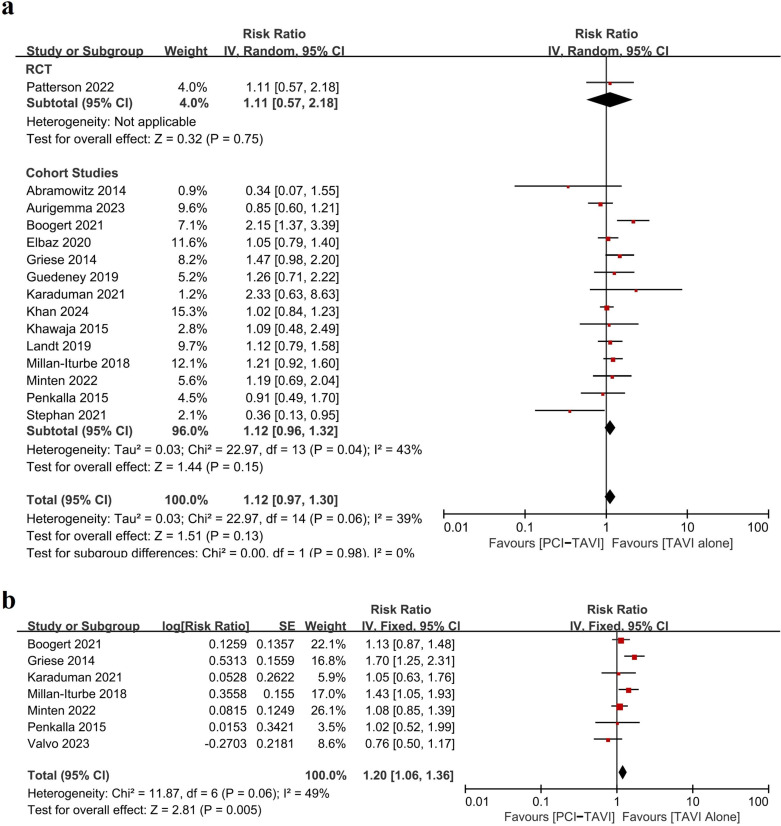
Forest plot of all-cause mortality at mid-term (a) or long-term (b) of patients undergoing PCI-TAVI versus TAVI alone. Studies were stratified by study type. Abbreviations: CI=confidence interval. RCT=randomized controlled trial. TAVI=transcatheter aortic valve implantation. PCI=percutaneous coronary intervention.

In studies comparing PCI-TAVI vs. TAVI alone, all-cause mortality was reported in 18 studies, 14 studies and 7 studies for short-term, mid-term and long-term follow-up respectively. There was no statistically significant difference between the two groups in short-term and mid-term follow-up (RR_short-term_ = 1.10, 95%CI 0.88-1.38, p = 0.39; RR_mid-term_ = 1.12, 95%CI 0.97-1.30, p = 0.13) (Fig 3 and 4), but in the long-term follow-up, patients undergoing PCI-TAVI had higher incidence of all-cause mortality than TAVI alone (RR_long-term_ = 1.20 95%CI 1.06-1.36 p = 0.005) (Fig 4).

We conducted subgroup analyses based on the type of THV. The results indicated that the all-cause mortality rate was not associated with the type of THV at short-term and long-term (S2 and S4 Tables). However, in the mid-term, PCI-TAVI group had a slightly higher incidence of all-cause mortality than TAVI alone in SEV > 20% subgroup (RR = 1.26, 95%CI 1.01-1.57, p = 0.04) (S3 Table).

**Fig 5:**
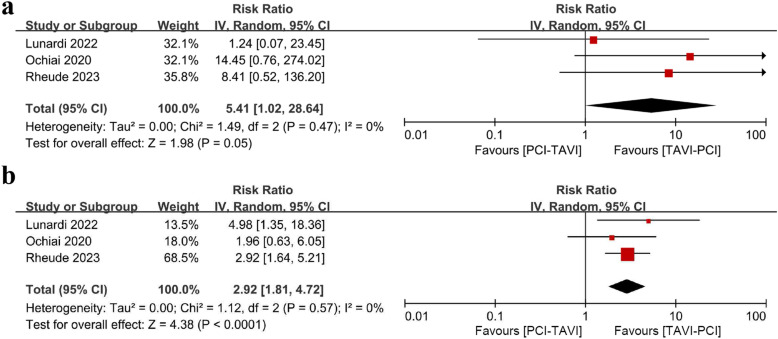
Forest plot of all-cause mortality at short-term (a) or long-term (b) of patients undergoing TAVI-PCI versus PCI-TAVI. Abbreviations: CI=confidence interval. RCT=randomized controlled trial. TAVI=transcatheter aortic valve implantation. PCI=percutaneous coronary intervention.

In studies comparing PCI-TAVI vs. TAVI-PCI, three studies reported all-cause mortality. Both in the short-term and long-term follow-up, all-cause mortality rate was lower in TAVI-PCI group compared to PCI-TAVI group (RR_short-term_, 5.41, 95% CI: 1.02-28.64, *P* =  0.05; RR_long-term_, 2.92, 95% CI: 1.81-4.72, *P* <  0.0001) (Fig 5).

#### Other individual events.

**Fig 6:**
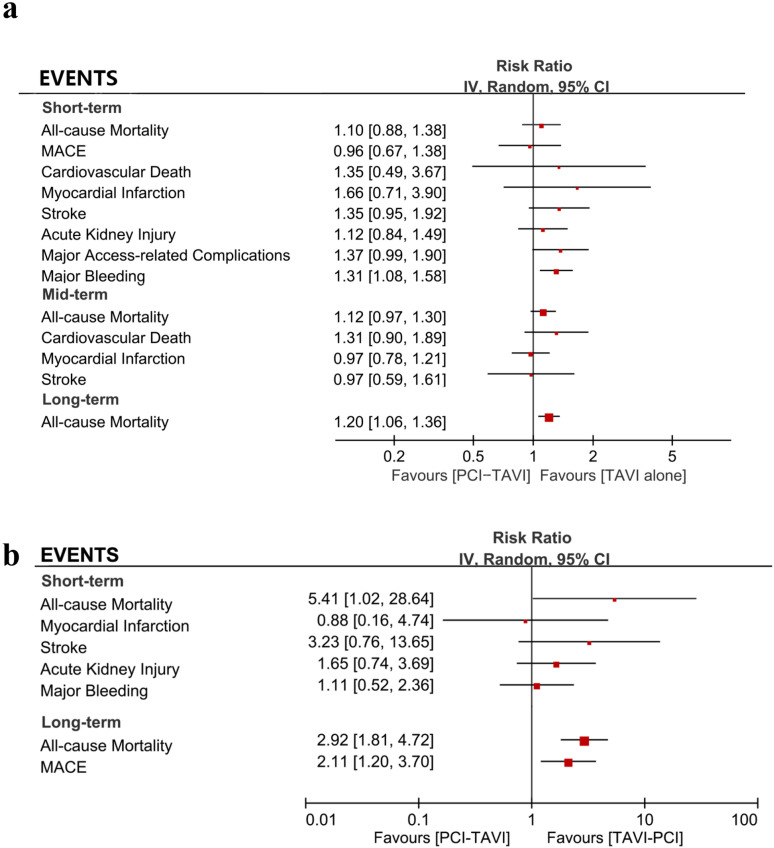
A summary of the rates for other individual events. a. Comparisons between PCI-TAVI vs. TAVI alone. b. Comparisons between PCI-TAVI vs. TAVI-PCI.

A summary of the rates for other individual events at short, mid, and long-term follow-up of PCI-TAVI vs. TAVI is shown in Table 3 and Fig 6. In the short-term, the incidence of major bleeding was higher in PCI-TAVI group than TAVI alone group. Detailed outcome of other individual events was displayed in S1 and S2 Figs.

**Table 3:** Overview of outcomes.

Outcome	Number of Studies	Effect Estimate (Risk Ratio [95% confidence interval])
PCI before/concomitant with TAVI vs. TAVI alone		
**Short-term (30d)**		
All-cause Mortality	18	1.10 [0.88, 1.38]
MACE	4	0.96 [0.67, 1.38]
Cardiovascular Death	4	1.35 [0.49, 3.67]
Myocardial Infarction	8	1.66 [0.71, 3.90]
Stroke	8	1.35 [0.95, 1.92]
Major Bleeding	11	1.31 [1.08, 1.58]
Acute Kidney Injury	7	1.12 [0.84, 1.49]
Major Access-related Complications	8	1.37 [0.99, 1.90]
Mid-term (6 months-1 year)		
All-cause Mortality	15	1.12 [0.97, 1.30]
Cardiovascular Death	6	1.31 [0.90, 1.89]
Myocardial Infarction	5	0.97 [0.78, 1.21]
Stroke	4	0.97 [0.59, 1.61]
**Long-term (2 years or longer)**		
All-cause Mortality	7	1.20 [1.06, 1.36]
**PCI before/concomitant with TAVI vs. PCI after TAVI**		
**Short-term (30d)**		
All-cause Mortality	3	5.41 [1.02, 28.64]
Myocardial Infarction	3	0.88 [0.16, 4.74]
Stroke	3	3.23 [0.76, 13.65]
Acute Kidney Injury	3	1.65 [0.74, 3.69]
Major Bleeding	3	1.11 [0.52, 2.36]
**Long-term (2 years or longer)**		
All-cause Mortality	3	2.92 [1.81, 4.72]
MACE	3	2.11 [1.20, 3.70]

Abbreviations: PCI=percutaneous coronary intervention. TAVI=transcatheter aortic valve implantation. MACE=major adverse cardiovascular events.

In the comparison of PCI-TAVI vs. TAVI-PCI, MACE at long-term follow-up is more frequent for patients undergoing PCI-TAVI as compared with TAVI-PCI (RR =  2.11, 95%CI 1.20-3.70). Detailed outcome of other individual events was displayed in Fig 6 and S3.

### Publication bias and sensitivity analysis

For outcome of all-cause mortality, funnel plot showed asymmetry (S4 Fig). We introduced Egger’s test (S5 Fig) which indicated no publication bias in short-term outcome (*p* =  0.313), mid-term (*p* =  0.880) or long-term *(p* = 0.522). We conducted a sensitivity analysis on the outcomes comparing PCI-TAVI to TAVI alone. The overall stability of the study results is satisfactory. Only in the long-term, the omitting of one study (Griese2014) influenced the overall outcome (S5 Table).

## Discussion

In this study, we summarized the current clinical studies on PCI timing strategies in patient undergoing TAVI. We discovered that PCI either before or concomitant with TAVI did not enhance short-term or medium-term clinical prognoses and correlated with increased long-term mortality. PCI after TAVI resulted in notably reduced all-cause mortality rates when contrasted with PCI before or concomitant with TAVI, irrespectively in the short or long-term.

In patients undergoing TAVI with concomitant CAD, many factors need consideration when determining whether to perform PCI and which timing strategy to adopt, including the degree of myocardial ischemia, the anatomy of the coronary arteries and aortic ostium, and bleeding risk. Current clinical guidelines focuses mainly on the location and stenosis degree of atherosclerotic plaques, recommending that PCI should be considered in patients with a primary indication to undergo TAVI and coronary artery diameter stenosis > 70% in proximal segments, with recommendation class Ⅱa, based on evidence level C [4]. One of the main rationales for conducting PCI prior to TAVI is the potential obstruction of the coronary ostium after THV deployment, complicating PCI procedures. In scenarios where patients necessitate immediate PCI due to acute myocardial infarction, there’s a risk of rescue delays and heightened mortality. Current data suggests that the success rate for coronary angiography post-SEV implantation is relatively lower compared to BEV. The elongated stent platform of SEV extends above the coronary ostium, necessitating the catheter to navigate through its diamond-shaped cell framework to conduct coronary angiography or PCI. Yet, overall, post-TAVI PCI exhibits a commendably high success rate [36]. Current studies indicate that the mean duration for executing PCI in post-TAVI patients stands at 52–58 minutes [36, 37], not exceeding the typical patient timeframe. Moreover, at present, there’s an absence of direct proof suggesting that TAVI-induced PCI postponements escalate mortality rates [38]. Furthermore, new “commissural alignment” techniques have been developed for performing PCI after TAVI, which could reduce the incidence of severe overlap of the coronary artery with the THV commissures [39, 40]. These techniques will further enhance the success rate of PCI after TAVI.

In this study, we did not find any prognostic improvement in patients undergoing PCI before or concomitant with TAVI. On the other hand, the long-term all-cause mortality rate is lower in both the TAVI alone group and TAVI-PCI group compared to the PCI-TAVI group. We attribute this outcome to the adherence to PCI indications, which facilitated the use of more appropriate antithrombotic strategies, thereby improving prognosis. We advocate that even for patients undergoing TAVI, the decision on PCI strategy should adhere to PCI indications rather than solely on the extent of stenosis. Current evidence indicates that PCI offers clinical prognostic benefits in the ACS population [41], but not significantly in the stable CAD population [6]. In the included studies, most participants who underwent PCI were diagnosed with CAD through coronary angiography or coronary CT, not clinically classified under ACS. This underscores the reason PCI-TAVI doesn’t enhance prognosis compared to TAVI alone. Compared with TAVI, PCI requires more intense antithrombotic treatment. A considerable proportion of stable CAD patients undergoing TAVI are compelled to adopt more aggressive antithrombotic therapy due to prior PCI. Therefore, the incidence of bleeding events in the PCI-TAVI group surpasses that of the TAVI alone group, potentially contributing to the worse outcomes observed in the PCI-TAVI group in the long-term. On the other hand, The TAVI-PCI strategy significantly improves prognosis compared to PCI-TAVI. We hypothesize several contributing factors. First, as previously discussed, after TAVI, ischemic events are the primary cause for subsequent PCI, which aligns with PCI indications. Second, successful treatment of AS eliminates left ventricular pressure overload and microvascular dysfunction and permits adequate physiological assessment of coronary lesion severity and identification of patients deriving benefit from revascularization. This could yield substantial benefits both in the periprocedural 30 days and over the long-term follow-up.

Owing to the symptom overlap between severe AS and severe CAD, it becomes difficult to identify coronary lesions with PCI indications based merely on clinical symptoms, particularly in patients not presenting with myocardial infarction. Pertaining to this, the FAME series of studies have demonstrated the clinical prognostic value of FFR-directed PCI [42]. Theoretically, this conclusion can be extrapolated to patients with AS combined with CAD to ascertain objective evidence of vascular ischemia. We believe that FFR-guided PCI strategies may provide benefits for these patients. According to current clinical guidelines, all patients undergoing TAVI are required to undergo CT angiography of the ascending aorta and coronary arteries. This allows for easy assessment of coronary artery stenosis via CT imaging. Furthermore, CT-derived FFR technology has shown growing diagnostic and prognostic value. However, evidence supporting PCI treatment guided by CT-derived FFR remains limited [43]. Therefore, we recommend performing coronary angiography, with FFR assessment if necessary, in patients with moderate or severe stenosis of the proximal coronary artery as identified by coronary CT angiography, prior to undergoing TAVI.

Compared to prior studies, we encompass a broader range of evidence, notably with significant updates in this domain in the recent 3 years. Similar to previous meta-analysis results, this study found no significant difference between PCI-TAVI and TAVI alone in the short- and mid-term. Pertaining to long-term outcomes (2 years or longer), prior meta-analyses hadn’t ventured into this territory, yet we identified significant differences between the two groups. In terms of safety outcomes, we found a significant increase in the risk of major bleeding. This finding is consistent with previous studies [44]. Methodologically, different from previous studies [45], we exclusively excluded studies involving patients without CAD. Our main concern was that certain retrospective studies included non-CAD patients in the TAVI alone group, who did not comply with indications for PCI, thus introducing bias. Consequently, this study excluded such studies to improve the reliability of the findings. Moreover, our work represents the first meta-analysis comparing PCI before or concomitant with TAVI versus PCI after TAVI, a comparison not explored in other studies previously.

The significance of this study is that it provides evidence suggesting potential adverse long-term outcomes associated with PCI-TAVI. Currently, a gap concerning the long-term prognosis of PCI-TAVI still exists. This research provides a robust addition to the existing clinical guidelines. Additionally, we collated current evidence highlighting the improved prognosis for PCI after TAVI compared with other timing strategies, further underscoring the significance of PCI strategy in line with established indications. However, additional high-quality evidence is required to confirm the superiority of post-TAVI PCI regarding long-term outcomes.

This study has several limitations: Firstly, we did not achieve analyses stratified by SEV or BEV in this study-level review. BEV is designed with a more concise stent platform, potentially mitigating coronary ostium obstruction in theory. Regarding SEV, there are notable structural differences among different brands of SEV, which influence the physician’s choice of treatment strategy. Furthermore, the impact of commissural alignment technology on clinical outcomes remains unclear. Yet, our subgroup analysis roughly revealed negligible disparities between SEV and BEV. Secondly, the severity of coronary stenosis and location atherosclerotic lesions may influence the prognosis of patients and are factors influencing PCI strategy. However, in this study, subgroup analysis based on patient SYNTAX scores and lesion location was not applicable because raw data were not given in original articles. Next, most of the studies included in this study are cohort studies, and not all studies were adjusted by statistics (propensity score matching, multivariable Cox regression), so the baselines are not aligned. Theoretically, compared to the TAVI alone group, the PCI-TAVI group has more severe coronary lesions and a worse prognosis. This study cannot rule out the possibility of biases.

## Conclusion

In this study, we conclude that PCI before or concomitantly with TAVI may not yield clinical benefits and could potentially result in a worse outcome in the long-term; PCI after TAVI is associated with improved clinical outcomes in both short- and long-term. We advocate that the decision to perform PCI should adhere to the established indications for PCI, instead of whether TAVI is being conducted.

## Supporting information

S1 Fig Individual events at 30 days. (a) Major adverse cardiovascular events. (b) Cardiovascular death. (c) non-fatal myocardial infarction. (d) Stroke.
(TIF)

S2 Fig Safety outcomes 30 days. (a) Major Bleeding. (b) Acute Kidney Injury. (c) Major Access-related Complications.
(TIF)

S3 Fig PCI-TAVI vs TAVI-PCI, other outcomes at 30 days. (a)non-fatal myocardial infarction. (b)stroke. (c)acute kidney injury. (d)major bleeding.
(TIF)

S4 Fig Funnel Plot. (a) short-term (b) mid-term and (c) long-term.
(TIF)

S5 Fig STATA Publication Bias Analysis. (a) short-term (b) mid-term and (c) long-term.
(TIF)

S1 Table Search strategy. (DOCX)

S2 Table Subgroup analysis of all-cause mortality in the short-term stratified by THV type. (DOCX)

S3 Table Subgroup analysis of all-cause mortality in the mid-term stratified by THV type. (DOCX)

S4 Table Subgroup analysis of all-cause mortality in the long-term stratified by THV type. (DOCX)

S5 Table Sensitivity analysis for comparison of PCI+TAVI vs. TAVI alone. (DOCX)

S1 File PRISMA2020 checklist. (DOCX)

S2 File Descriptions of 813 records and exclusion reasons. (XLSX)

S3 File Minimal data set. (XLSX)
