# Supplementary figures and images for: Impact of PCI strategies on outcomes of patients undergoing Transcatheter Aortic Valve Implantation with concomitant coronary artery disease: A systematic review and meta-analysis
Source: PLoS One. 2025 Apr 30;20(4):e0321395. doi: 10.1371/journal.pone.0321395 (PMC12043176; doi:10.1371/journal.pone.0321395)

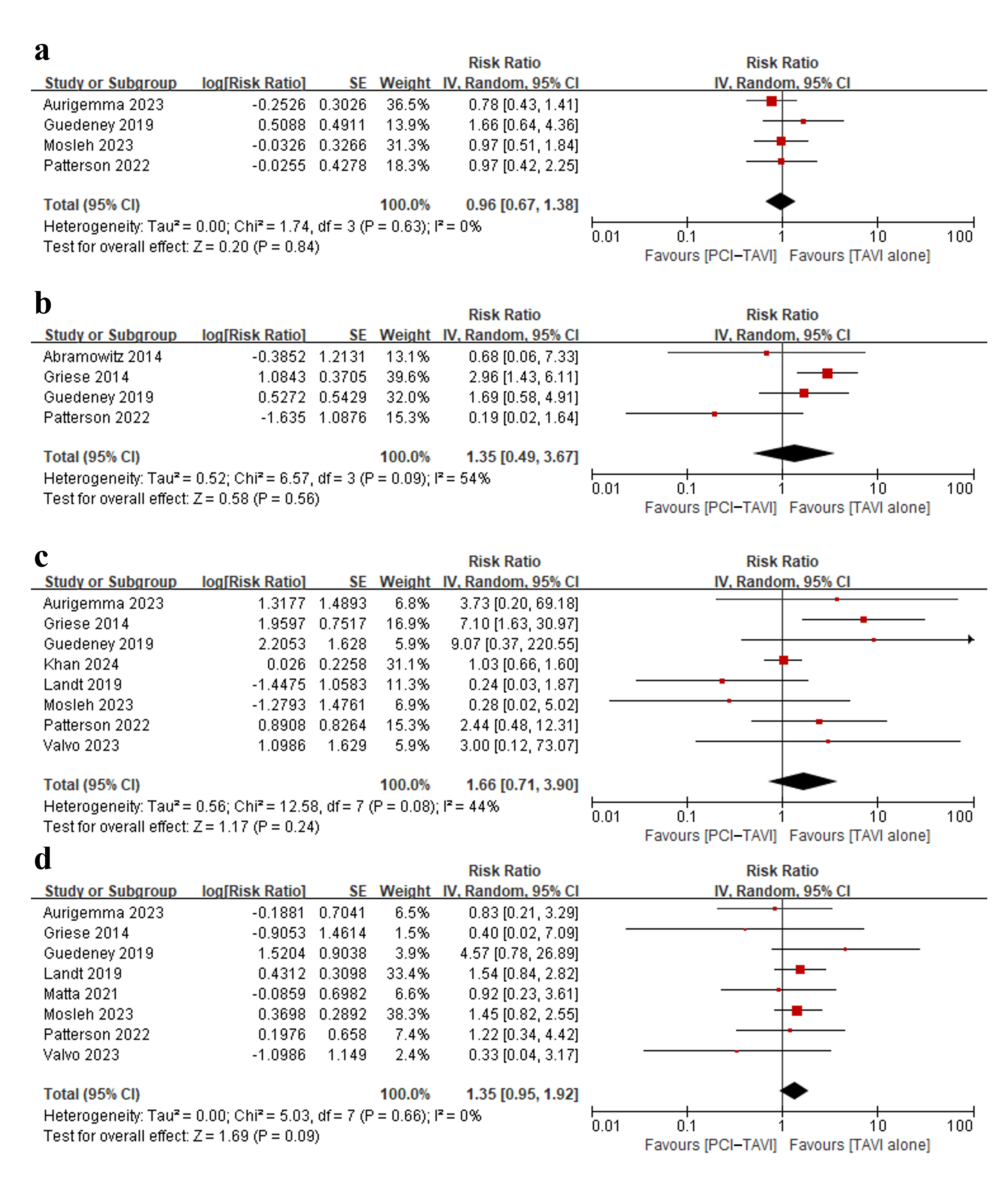

Supplement: S1 Fig — (a) Major adverse cardiovascular events. (b) Cardiovascular death. (c) non-fatal myocardial infarction. (d) Stroke. (TIF) [file pone.0321395.s001.tif]

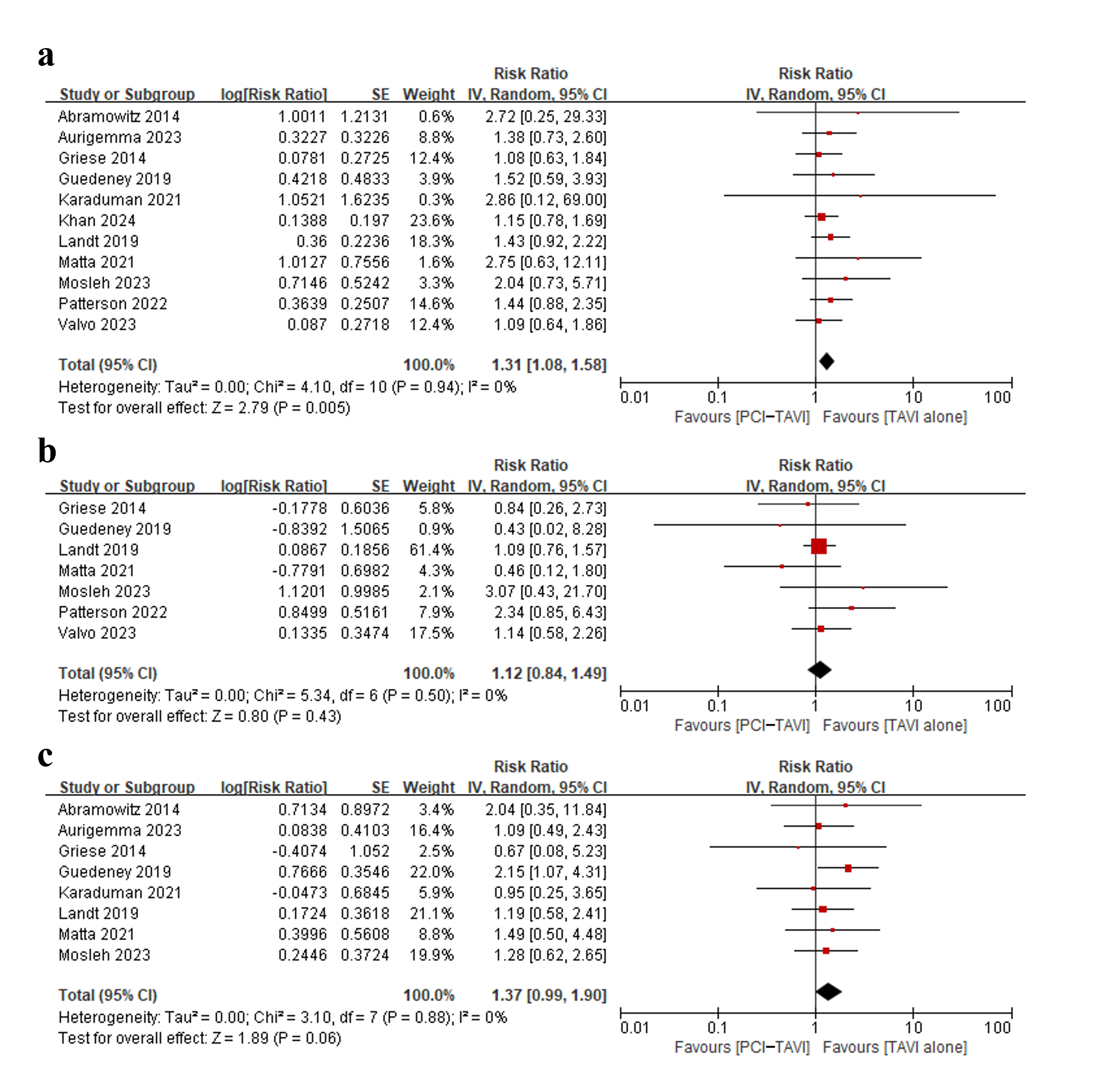

Supplement: S2 Fig — (a) Major Bleeding. (b) Acute Kidney Injury. (c) Major Access-related Complications. (TIF) [file pone.0321395.s002.tif]

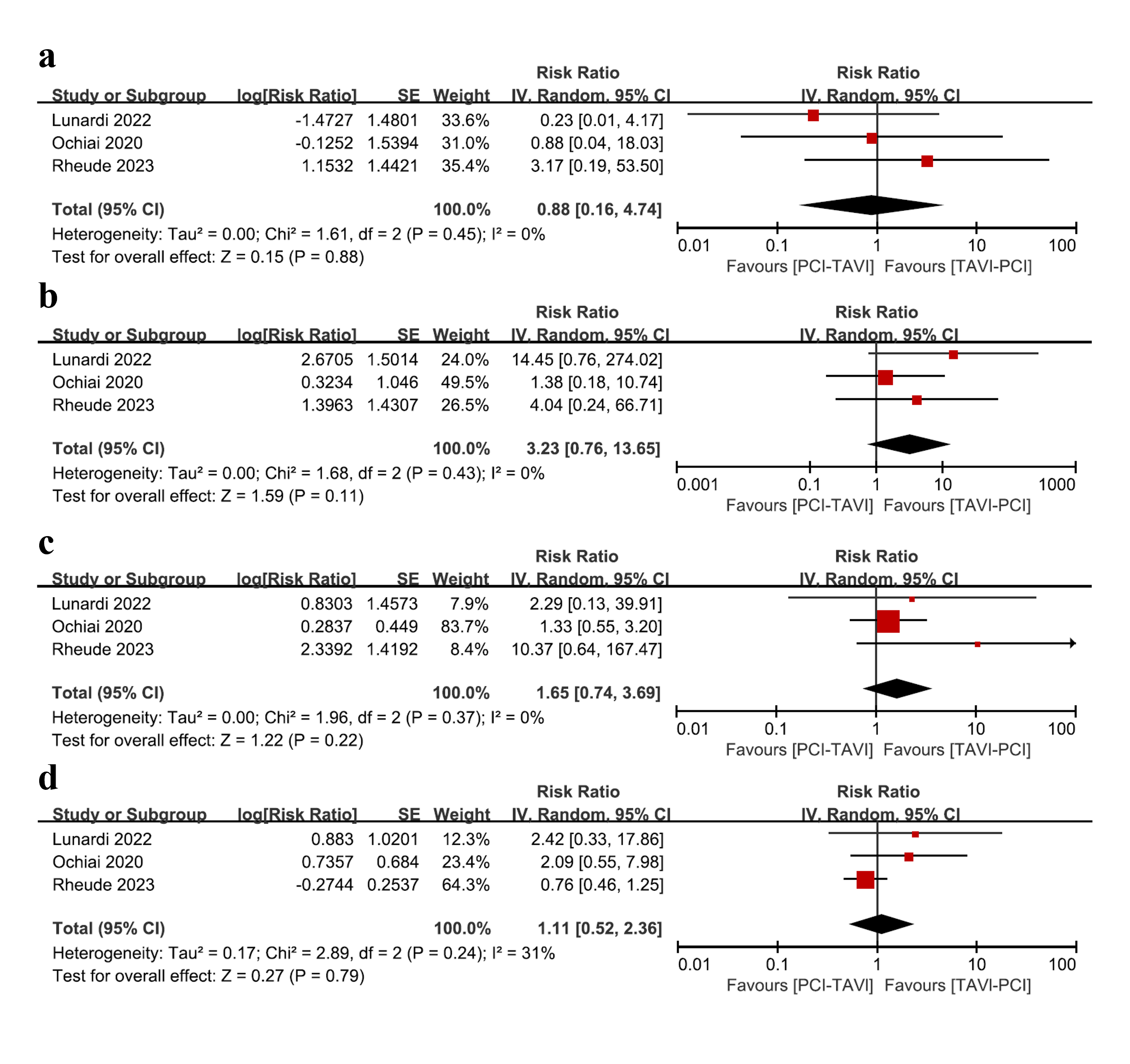

Supplement: S3 Fig — (a)non-fatal myocardial infarction. (b)stroke. (c)acute kidney injury. (d)major bleeding. (TIF) [file pone.0321395.s003.tif]

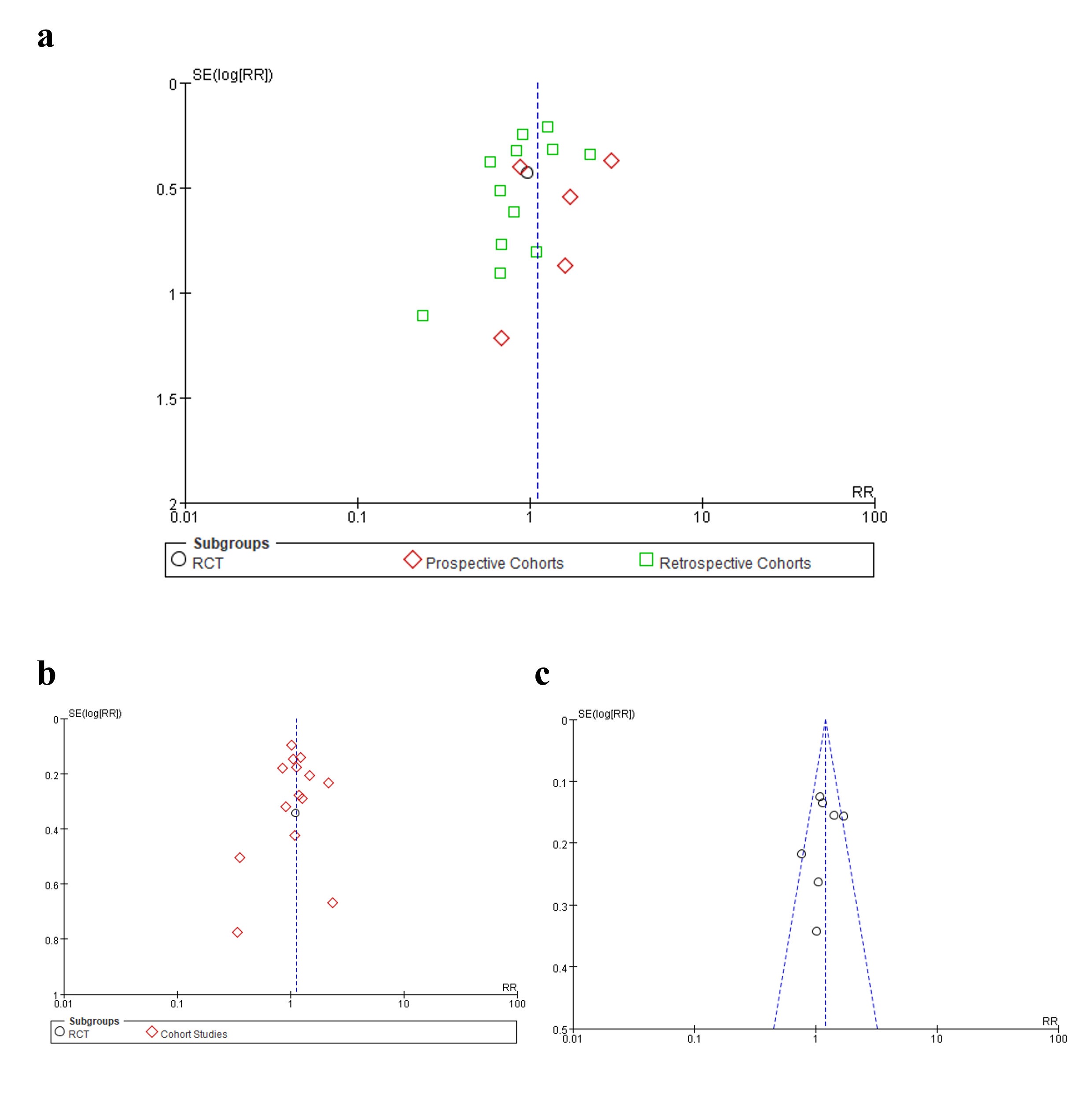

Supplement: S4 Fig — (a) short-term (b) mid-term and (c) long-term. (TIF) [file pone.0321395.s004.tif]

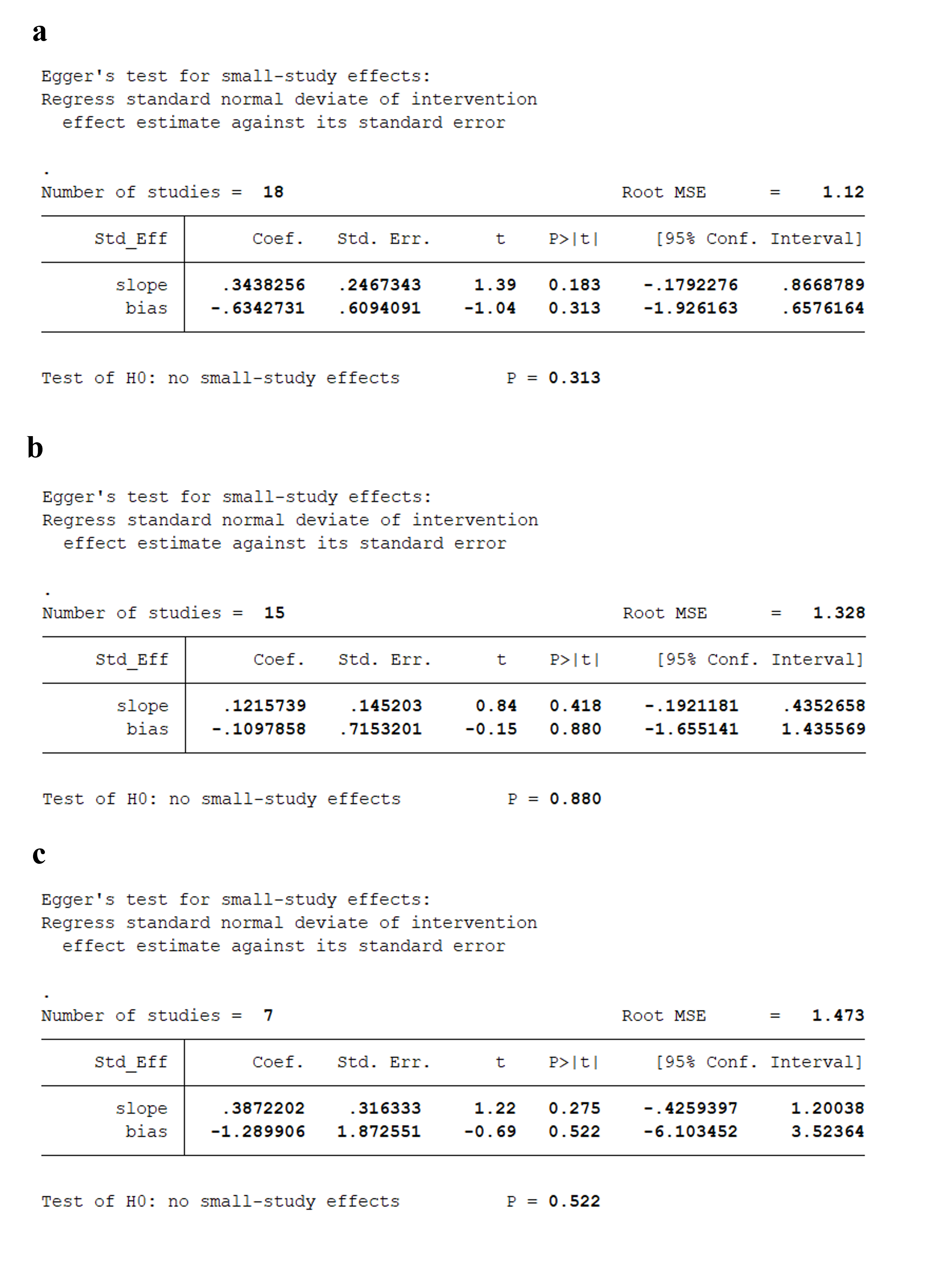

Supplement: S5 Fig — STATA Publication Bias Analysis. (a) short-term (b) mid-term and (c) long-term. (TIF) [file pone.0321395.s005.tif]
