# Supplementary material for: Impact of PCI strategies on outcomes of patients undergoing Transcatheter Aortic Valve Implantation with concomitant coronary artery disease: A systematic review and meta-analysis
Source: PLoS One. 2025 Apr 30;20(4):e0321395. doi: 10.1371/journal.pone.0321395 (PMC12043176; doi:10.1371/journal.pone.0321395)
Supplement: S1 Table — (DOCX) [file pone.0321395.s006.docx]

## S1 Table

| Table S1. Search strategy. | |
| --- | --- |
| Database | Search strategy |
| PubMed (n=470) | ((Transcatheter aortic valve implantation [Title/Abstract]) OR (Transcatheter aortic valve replacement [Title/Abstract]) OR (TAVI[Title/Abstract]) OR (TAVR[Title/Abstract]) NOT (surgical [Title]))  AND  ((Percutaneous Coronary Intervention [Title/Abstract]) OR (Revascularization [Title/Abstract]) OR (Percutaneous revascularization [Title/Abstract]) OR (Angioplasty [Title/Abstract]) OR (Stenting [Title/Abstract]) OR (PCI [Title/Abstract]) OR (PTCA [Title/Abstract]) OR (Coronary Artery Disease[Title/Abstract]))  AND  ((MACE[Title/Abstract]) OR (Major adverse cardiovascular events[Title/Abstract] ) OR (cardiovascular events[Title/Abstract] ) OR (outcomes[Title/Abstract] ) OR (prognosis[Title/Abstract] ) OR (AMI[Title/Abstract] ) OR (Myocardial infarction[Title/Abstract] ) OR (Mortality[Title/Abstract] ) OR (Death[Title/Abstract] ) OR (Rehospitalization[Title/Abstract] ) OR (Hospitalization[Title/Abstract] ) OR (readmission[Title/Abstract]))  NOT  ((Review[Filter]) OR (Meta-Analysis [Filter]) OR(Case Reports [Filter]) OR (Books and Documents [Filter]) OR (Editorial[Filter])) |
| EMBASE (n=356) | ((Transcatheter aortic valve implantation):ti,ab,kw OR (Transcatheter aortic valve replacement):ti,ab,kw OR (TAVI):ti,ab,kw OR (TAVR):ti,ab,kw NOT (surgical):TI) AND ((Percutaneous Coronary Intervention):ti,ab,kw OR (Revascularization):ti,ab,kw OR (Percutaneous revascularization):ti,ab,kw OR (Angioplasty):ti,ab,kw OR (Stenting):ti,ab,kw OR (PCI):ti,ab,kw OR (PTCA):ti,ab,kw )  AND ((MACE):ti,ab,kw OR (Major adverse cardiovascular events):ti,ab,kw OR (cardiovascular events):ti,ab,kw OR (outcomes):ti,ab,kw OR (prognosis):ti,ab,kw OR (AMI):ti,ab,kw OR (Myocardial infarction):ti,ab,kw OR (Mortality):ti,ab,kw OR (Death):ti,ab,kw OR (Rehospitalization):ti,ab,kw OR (Hospitalization):ti,ab,kw OR (Readmission):ti,ab,kw) AND ('Article'/it OR 'Article in Press'/it OR 'Conference Paper'/it) NOT ('case report'/de OR 'nonhuman'/de OR 'meta analysis'/de OR 'systematic review'/de) |
| Cochrane Library (n=79) | ((Transcatheter aortic valve implantation):ti,ab,kw OR (Transcatheter aortic valve replacement):ti,ab,kw OR (TAVI):ti,ab,kw OR (TAVR):ti,ab,kw NOT (surgical):ti) AND ((Percutaneous Coronary Intervention):ti,ab,kw OR (Revascularization):ti,ab,kw OR (Percutaneous revascularization):ti,ab,kw OR (Angioplasty):ti,ab,kw OR (Stenting):ti,ab,kw OR (PCI):ti,ab,kw OR (PTCA):ti,ab,kw ) AND ((MACE):ti,ab,kw OR (Major adverse cardiovascular events):ti,ab,kw OR (cardiovascular events):ti,ab,kw OR (outcomes):ti,ab,kw OR (prognosis):ti,ab,kw OR (AMI):ti,ab,kw OR (Myocardial infarction):ti,ab,kw OR (Mortality):ti,ab,kw OR (Death):ti,ab,kw OR (Rehospitalization):ti,ab,kw OR (Hospitalization):ti,ab,kw OR (Readmission):ti,ab,kw) |
| Web of Science (n=617) | #1 TS=(Transcatheter aortic valve implantation OR Transcatheter aortic valve replacement OR TAVI OR TAVR)  #2 TI=surgical  #3 TS=(Percutaneous Coronary Intervention OR Revascularization OR Percutaneous revascularization OR Angioplasty OR Stenting OR PCI OR PTCA )  #4 TS=(MACE OR Major adverse cardiovascular events OR cardiovascular events OR outcomes OR prognosis OR AMI OR myocardial infarction OR Mortality OR Death OR Rehospitalization OR Hospitalization OR Readmission)  #5 DT==("ARTICLE")  #6 SILOID==("PPRN") OR DT==("REVIEW" OR "MEETING" OR "CASE REPORT" OR "EDITORIAL MATERIAL" OR "REFERENCE MATERIAL" OR "ABSTRACT" OR "LETTER" OR "NEWS")  #7 (#1 NOT #2) AND #3 AND #4 AND #5 NOT #6 |
|  | |
