# Supplementary material for: Impact of PCI strategies on outcomes of patients undergoing Transcatheter Aortic Valve Implantation with concomitant coronary artery disease: A systematic review and meta-analysis
Source: PLoS One. 2025 Apr 30;20(4):e0321395. doi: 10.1371/journal.pone.0321395 (PMC12043176; doi:10.1371/journal.pone.0321395)
Supplement: S2 Table — (DOCX) [file pone.0321395.s007.docx]

## Table S2

| Table S2. Subgroup analysis of all-cause mortality in the short-term stratified by THV type | | | |
| --- | --- | --- | --- |
| Subgroup and Study ID | Log RR | SE | RR [95%CI] |
| **SEV<20%** |  |  |  |
| Patterson 2022 | -0.0255 | 0.4278 | 0.97 [0.42, 2.25] |
| Boogert 2021 | 0.7948 | 0.3435 | 2.21 [1.13, 4.34] |
| Griese 2014 | 1.0843 | 0.3705 | 2.96 [1.43, 6.11] |
| Karaduman 2021 | -1.4335 | 1.1039 | 0.24 [0.03, 2.08] |
| Khawaja 2015 | 0.0843 | 0.8033 | 1.09 [0.23, 5.25] |
| Penkalla 2015 | -0.3881 | 0.7705 | 0.68 [0.15, 3.07] |
| **Subtotal (95% CI)** |  |  | **1.41 [0.78, 2.54]** |
| **SEV>20%** |  |  |  |
| Elbaz 2020 | -0.0984 | 0.2474 | 0.91 [0.56, 1.47] |
| Guedeney 2019 | 0.5272 | 0.5429 | 1.69 [0.58, 4.91] |
| Huczek 2018 | -0.1806 | 0.3235 | 0.83 [0.44, 1.57] |
| Landt 2019 | -0.5382 | 0.3749 | 0.58 [0.28, 1.22] |
| Mancio 2015 | 0.4689 | 0.8692 | 1.60 [0.29, 8.78] |
| Matta 2021 | -0.2195 | 0.6168 | 0.80 [0.24, 2.69] |
| Mosleh 2023 | 0.2934 | 0.3158 | 1.34 [0.72, 2.49] |
| **Subtotal (95% CI)** |  |  | **0.95 [0.72, 1.26]** |
| **NR** |  |  |  |
| Abramowitz 2014 | -0.3852 | 1.2131 | 0.68 [0.06, 7.33] |
| Stephan 2021 | -0.4055 | 0.9021 | 0.67 [0.11, 3.91] |
| Valvo 2023 | -0.4055 | 0.5143 | 0.67 [0.24, 1.83] |
| Aurigemma 2023 | -0.134 | 0.3993 | 0.87 [0.40, 1.91] |
| Khan 2024 | 0.2283 | 0.2123 | 1.26 [0.83, 1.90] |
| **Subtotal (95% CI)** |  |  | 1.06 [0.76, 1.48] |
| **Total (95% CI)** |  |  | **1.10 [0.88, 1.38]** |
| Abbreviations: THV, transcatheter heart valve. SEV, self-expanding valve. NR, not reported. | | | |
