# Supplementary material for: Impact of PCI strategies on outcomes of patients undergoing Transcatheter Aortic Valve Implantation with concomitant coronary artery disease: A systematic review and meta-analysis
Source: PLoS One. 2025 Apr 30;20(4):e0321395. doi: 10.1371/journal.pone.0321395 (PMC12043176; doi:10.1371/journal.pone.0321395)
Supplement: S3 Table — (DOCX) [file pone.0321395.s008.docx]

## Table S3

| Table S3. Subgroup analysis of all-cause mortality in the mid-term stratified by THV type | | | |
| --- | --- | --- | --- |
| Subgroup and Study ID | Log RR | SE | RR [95%CI] |
| **SEV<20%** |  |  |  |
| Griese 2014 | 0.3858 | 0.2062 | 1.47 [0.98, 2.20] |
| Khawaja 2015 | 0.0843 | 0.4226 | 1.09 [0.48, 2.49] |
| Penkalla 2015 | -0.0953 | 0.3183 | 0.91 [0.49, 1.70] |
| Karaduman 2021 | 0.8473 | 0.6674 | 2.33 [0.63, 8.63] |
| Patterson 2022 | 0.108 | 0.3419 | 1.11 [0.57, 2.18] |
| **Subtotal (95% CI)** |  |  | **1.26 [0.95, 1.66]** |
| **SEV>20%** |  |  |  |
| Millan-Iturbe 2018 | 0.1933 | 0.1401 | 1.21 [0.92, 1.60] |
| Guedeney 2019 | 0.2276 | 0.2906 | 1.26 [0.71, 2.22] |
| Landt 2019 | 0.113 | 0.1773 | 1.12 [0.79, 1.58] |
| Elbaz 2020 | 0.052 | 0.1466 | 1.05 [0.79, 1.40] |
| Boogert 2021 | 0.7674 | 0.2318 | 2.15 [1.37, 3.39] |
| **Subtotal (95% CI)** |  |  | **1.26 [1.01, 1.57]** |
| **NR** |  |  |  |
| Abramowitz 2014 | -1.0783 | 0.7724 | 0.34 [0.07, 1.55] |
| Stephan 2021 | -1.0296 | 0.5018 | 1.19 [0.69, 2.04] |
| Minten 2022 | 0.1711 | 0.2767 | 0.99 [0.72, 1.36] |
| Aurigemma 2023 | -0.164 | 0.1789 | 0.36 [0.13, 0.95] |
| Khan 2024 | 0.0158 | 0.0978 | 1.02 [0.84, 1.23] |
| **Subtotal (95% CI)** |  |  | **0.89 [0.67, 1.18]** |
| **Total (95% CI)** |  |  | **1.12 [0.97, 1.30]** |
| Abbreviations: THV, transcatheter heart valve. SEV, self-expanding valve. NR, not reported. | | | |
