# Supplementary material for: Impact of PCI strategies on outcomes of patients undergoing Transcatheter Aortic Valve Implantation with concomitant coronary artery disease: A systematic review and meta-analysis
Source: PLoS One. 2025 Apr 30;20(4):e0321395. doi: 10.1371/journal.pone.0321395 (PMC12043176; doi:10.1371/journal.pone.0321395)
Supplement: S4 Table — (DOCX) [file pone.0321395.s009.docx]

## Table S4

| Table S4. Subgroup analysis of all-cause mortality in the long-term stratified by THV type | | | |
| --- | --- | --- | --- |
| Subgroup and Study ID | Log RR | SE | RR [95%CI] |
| **SEV<20%** |  |  |  |
| Boogert 2021 | 0.1259 | 0.1357 | 1.13 [0.87, 1.48] |
| Griese 2014 | 0.5313 | 0.1559 | 1.70 [1.25, 2.31] |
| Karaduman 2021 | 0.0528 | 0.2622 | 1.05 [0.63, 1.76] |
| Penkalla 2015 | 0.0153 | 0.3421 | 1.02 [0.52, 1.99] |
| **Subtotal (95% CI)** |  |  | 1.26 [0.98, 1.63] |
| **SEV>20%** |  |  |  |
| Millan-Iturbe 2018 | 0.3558 | 0.155 | 1.43 [1.05, 1.93] |
| **Subtotal (95% CI)** |  |  | **1.43 [1.05, 1.93]** |
| **NR** |  |  |  |
| Minten 2022 | 0.4011 | 0.1294 | 1.49 [1.16, 1.92] |
| Valvo 2023 | -0.2703 | 0.2181 | 0.76 [0.50, 1.17] |
| **Subtotal (95% CI)** |  |  | 1.09 [0.57, 2.11] |
| **Total (95% CI)** |  |  | **1.25 [1.03, 1.52]** |
| Abbreviations: THV, transcatheter heart valve. SEV, self-expanding valve. NR, not reported. | | | |
