# Supplementary material for: Impact of PCI strategies on outcomes of patients undergoing Transcatheter Aortic Valve Implantation with concomitant coronary artery disease: A systematic review and meta-analysis
Source: PLoS One. 2025 Apr 30;20(4):e0321395. doi: 10.1371/journal.pone.0321395 (PMC12043176; doi:10.1371/journal.pone.0321395)
Supplement: S5 Table — (DOCX) [file pone.0321395.s010.docx]

## Table S5

| Table S5. Sensitivity analysis for comparison of PCI+TAVI vs. TAVI alone | |
| --- | --- |
| Study omitted | Estimate RR and 95%Confidence Interval |
| **Short-term** | |
| Patterson2022 | 1.11 [0.87, 1.41] |
| Abramowitz2014 | 1.11 [0.88, 1.40] |
| Boogert2021 | 1.05 [0.85, 1.29] |
| Elbaz2020 | 1.13 [0.88, 1.45] |
| Griese2014 | 1.04 [0.86, 1.27] |
| Guedeney2019 | 1.08 [0.86, 1.37] |
| Huczek2018 | 1.13 [0.89, 1.44] |
| Karaduman2021 | 1.12 [0.90, 1.40] |
| Khawaja2015 | 1.10 [0.87, 1.39] |
| Landt2019 | 1.17 [0.94, 1.45] |
| Mancio2015 | 1.10 [0.87, 1.38] |
| Matta2021 | 1.11 [0.88, 1.41] |
| Penkalla2015 | 1.11 [0.88, 1.41] |
| Stephan2021 | 1.11 [0.88, 1.40] |
| Valvo2023 | 1.13 [0.90, 1.42] |
| Aurigemma2023 | 1.12 [0.88, 1.42] |
| Mosleh2023 | 1.08 [0.85, 1.38] |
| Khan2024 | 1.08 [0.83, 1.39] |
| **Mid-term** | |
| Patterson2022 | 1.12 [0.96, 1.32] |
| Abramowitz2014 | 1.13 [0.98, 1.31] |
| Boogert2021 | 1.07 [0.95, 1.20] |
| Elbaz2020 | 1.13 [0.96, 1.34] |
| Griese2014 | 1.10 [0.94, 1.28] |
| Guedeney2019 | 1.12 [0.95, 1.31] |
| Karaduman2021 | 1.11 [0.96, 1.29] |
| Khawaja2015 | 1.12 [0.96, 1.31] |
| Landt2019 | 1.12 [0.95, 1.33] |
| Millan-Iturbe 2018 | 1.11 [0.94, 1.32] |
| Minten 2022 | 1.12 [0.95, 1.31] |
| Penkalla 2015 | 1.13 [0.97, 1.33] |
| Stephan 2021 | 1.14 [1.00, 1.31] |
| Aurigemma2023 | 1.16 [0.99, 1.35] |
| Khan2024 | 1.14 [0.96, 1.36] |
| **Long-term** | |
| Millan-Iturbe2018 | 1.15 [1.01, 1.32] |
| Minten2022 | 1.24 [1.07, 1.43] |
| Penkalla2015 | 1.20 [1.06, 1.37] |
| Boogert2021 | 1.21 [1.05, 1.40] |
| Griese2014* | 1.11 [0.97, 1.28] |
| Karaduman2021 | 1.21 [1.06, 1.37] |
| Valvo2023 | 1.25 [1.09, 1.42] |
| *Omitting the study affects the stability of total results. Abbreviations: RR=risk ratio. | |
